# Human-to-Human Transmission of Influenza A(H3N2) Virus with Reduced Susceptibility to Baloxavir, Japan, February 2019

**DOI:** 10.3201/eid2511.190757

**Published:** 2019-11

**Authors:** Emi Takashita, Masataka Ichikawa, Hiroko Morita, Rie Ogawa, Seiichiro Fujisaki, Masayuki Shirakura, Hideka Miura, Kazuya Nakamura, Noriko Kishida, Tomoko Kuwahara, Hiromi Sugawara, Aya Sato, Miki Akimoto, Keiko Mitamura, Takashi Abe, Masahiko Yamazaki, Shinji Watanabe, Hideki Hasegawa, Takato Odagiri

**Affiliations:** National Institute of Infectious Diseases, Tokyo, Japan (E. Takashita, H. Morita, R. Ogawa, S. Fujisaki, M. Shirakura, H. Miura, K. Nakamura, N. Kishida, T. Kuwahara, H. Sugawara, A. Sato, M. Akimoto, S. Watanabe, H. Hasegawa, T. Odagiri);; Ichikawa Children’s Clinic, Kanagawa, Japan (M. Ichikawa); Eiju General Hospital, Tokyo (K. Mitamura);; Abe Children’s Clinic, Kanagawa (T. Abe);; Zama Children’s Clinic, Kanagawa (M. Yamazaki)

**Keywords:** influenza virus, cap-dependent endonuclease inhibitor, baloxavir marboxil, baloxavir acid, antimicrobial resistance, human-to-human transmission, family cluster, H3N2, influenza A(H1N1)pdm09, baloxavir, Japan, viruses, respiratory infections, influenza, I38T substitution, polymerase acidic, whole-genome sequencing

## Abstract

In 2019, influenza A(H3N2) viruses carrying an I38T substitution in the polymerase acidic gene, which confers reduced susceptibility to baloxavir, were detected in Japan in an infant without baloxavir exposure and a baloxavir-treated sibling. These viruses’ whole-genome sequences were identical, indicating human-to-human transmission. Influenza virus isolates should be monitored for baloxavir susceptibility.

The cap-dependent endonuclease inhibitor baloxavir marboxil is approved in Japan for the treatment of influenza virus infection in patients >12 years of age and children <12 years of age weighing >10 kg. In phase 2 and 3 clinical trials of baloxavir, treatment-emergent amino acid substitutions––I38T or I38F for influenza A(H1N1)pdm09 (pH1N1) virus and I38T or I38M for influenza A(H3N2) virus in the polymerase acidic (PA) protein––were detected (*1*,*2*). The frequency of infections with these viruses was higher in patients <12 years of age than in those 12–64 years of age (*3*). Furthermore, PA I38 substitutions emerged more frequently in influenza A(H3N2) viruses than in pH1N1 virus or influenza B virus (*3*).

In phase 3 trials, patients infected with mutant viruses encoding the PA I38 substitution exhibited prolonged virus shedding, and the median time to symptom alleviation was longer in baloxavir recipients infected with these viruses than those infected with viruses not harboring these substitutions (*1*,*2*). Therefore, starting in the 2017–18 influenza season, we began monitoring baloxavir susceptibility of influenza viruses nationwide (*4*). In the 2018–19 season, we found that 1.5% (5/323) of pH1N1 and 9.5% (32/337) of H3N2 viruses possessed a PA I38 substitution (Table 1). All 5 pH1N1 viruses and 28 of 32 H3N2 viruses encoding a PA I38 substitution were recovered from patients after baloxavir administration. In January 2019, we detected a mutant influenza A(H3N2) virus carrying the PA I38T substitution from a hospitalized 5-year-old child who was not treated with baloxavir (*5*). We subsequently detected 3 similar mutant H3N2 viruses from 3 baloxavir-untreated children. Two of 3 were detected in sporadic cases and the other from a family cluster. Here, we report on the family cluster.

**Table 1:** Influenza viruses with I38 substitutions in polymerase acidic protein, Japan, 2018–19*

Influenza type or subtype	Total frequency	Age group, y				
		0–11	12–19	20–64	>65	Unknown
A(H1N1)pdm09	5/323 (1.5)	4/230 (1.7)	1/35 (2.9)	0/41	0/14	0/3
A(H3N2)	32/337 (9.5)	26/215 (12.1)	5/45 (11.1)	1/54 (1.9)	0/16	0/7
B	0/36	0/21	0/7	0/6	0/1	0/1

*Values are no./total (%).

## The Study

In February 2019, we detected 2 H3N2 viruses in siblings within a family cluster (Figure). The first child (a 10-year-old) experienced symptom onset on February 5 and was treated with baloxavir 12 hours later; this child’s fever resolved within a half day of baloxavir administration. The second child (an 8-month-old infant weighing <10 kg) experienced symptom onset on February 6 and received neuraminidase (NA) inhibitor oseltamivir 12 hours later; the infant’s fever resolved within 2 days of oseltamivir administration.

**Figure:**
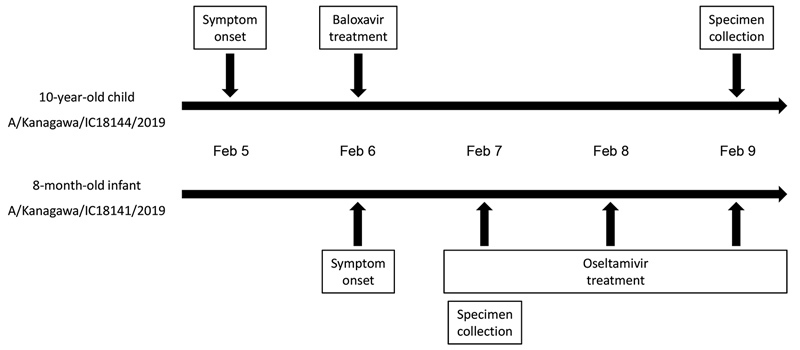
Clinical timeline of 2 siblings infected with mutant influenza A(H3N2) viruses encoding the polymerase acidic I38T substitution, Japan, February 2019. Whole-genome sequences of A/Kanagawa/IC18144/2019 (isolate no. EPI ISL 346656) and A/Kanagawa/IC18141/2019 (isolate no. EPI ISL 345215) are available from the GISAID EpiFlu database (http://www.gisaid.org).

We collected a nasal blow sample from the 10-year-old child 3 days after baloxavir administration and a nasal aspirate from the infant on the first day of oseltamivir administration. Deep sequencing analysis (*4*) of the virus isolates (A/Kanagawa/IC18144/2019 in 10-year-old and A/Kanagawa/IC18141/2019 in infant) with MiSeq (Illumina, https://www.illumina.com) revealed that the whole-genome sequences of these viruses were identical. Both viruses possessed the PA I38T substitution and did not contain wild-type 38I. No amino acid substitutions associated with reduced susceptibility to NA inhibitors were detected.

We determined the susceptibilities of this mutant virus to baloxavir acid (hydrolyzed active form; MedChemexpress, https://www.medchemexpress.com) and the 4 NA inhibitors approved for use in Japan: oseltamivir carboxylate (Sequoia Research Products, http://www.seqchem.com), peramivir (Sequoia Research Products), zanamivir (Sequoia Research Products), and laninamivir (Daiichi Sankyo, https://www.daiichisankyo.com). Because the genomic sequences of A/Kanagawa/IC18141/2019 and A/Kanagawa/IC18144/2019 were identical, we analyzed only A/Kanagawa/IC18141/2019. We determined antiviral susceptibilities by using a focus reduction assay and a fluorescent NA inhibition assay (NA-Fluor Influenza Neuraminidase Assay Kit; Applied Biosystems, https://www.thermofisher.com) (*4*) and calculated 50% inhibitory concentration (IC_50_) values using MikroWin 2010 (Labsis, https://labsis.de). To interpret the NA inhibitor susceptibility, we applied the World Health Organization criteria of IC_50_ fold-change values compared with reference IC_50_ values (*6*). The World Health Organization criteria define influenza A virus inhibition as normal (<10-fold increase), reduced (10–100-fold increase), or highly reduced (>100-fold increase).

The mutant virus encoding the PA I38T substitution showed normal inhibition with all 4 NA inhibitors but exhibited a 186-fold higher IC_50_ value (236 nmol/L) to baloxavir compared with the median IC_50_ value of influenza A(H3N2) viruses isolated in the 2018–19 season in Japan (1.27 nmol/L; Table 2). These results indicate that the mutant virus we isolated carrying the PA I38T substitution had reduced susceptibility to baloxavir but remained susceptible to NA inhibitors (*5*,*7*).

**Table 2:** Susceptibility of influenza A(H3N2) virus carrying polymerase acidic I38T substitution detected in children within family cluster, February 2019, compared with 2018–19 seasonal virus, Japan*

Influenza virus	Median IC_50_ + SD, nmol/L				
	Baloxavir	NA inhibitors (WHO criteria)			
		Oseltamivir	Peramivir	Zanamivir	Laninamivir
A/Kanagawa/IC18141/2019	236.08	0.37 (NI)	0.18 (NI)	1.01 (NI)	1.27 (NI)
A(H3N2) of 2018–19	1.27 + 1.08†	0.37 + 0.17‡	0.13 + 0.03‡	0.79 + 0.33‡	1.00 + 0.21‡

*We determined antiviral susceptibilities using a focus reduction assay and a fluorescent NA inhibition assay (NA-Fluor Influenza Neuraminidase Assay Kit; Applied Biosystems, https://www.thermofisher.com) (*4*) and calculated IC_50_ values using MikroWin 2010 (Labsis, https://labsis.de). We expressed NA inhibitor susceptibilities using WHO criteria for influenza A virus inhibition, which define susceptibility on the basis of the -fold change in IC_50_ compared with the IC_50_ of reference isolates (*6*). WHO inhibition was defined as normal (<10-fold increase), reduced (10–100-fold increase), or highly reduced (>100-fold increase) in comparison with the median value of isolates from the same influenza season. IC_50_, 50% inhibitory concentration; NA, neuraminidase; NI, normal inhibition; WHO, World Health Organization. †n = 83. ‡n = 170.

## Conclusions

During the 2018–19 influenza season in Japan, we detected 32 mutant influenza A(H3N2) viruses carrying various types of PA I38 substitutions, 4 of which were isolated from children <12 years of age without prior baloxavir exposure. Almost all mutant viruses isolated from baloxavir-treated patients possessed mixed PA I38T/I, I38M/I, I38R/I, I38T/M/I, I38T/K/I, or I38T/M/R substitutions (*5*), indicating these mutant viruses emerged under the selective pressure of baloxavir. In contrast, the 4 mutant viruses recovered from children without prior baloxavir treatment, including the virus described in this study, contained the PA I38T substitution and not a mixture including wild-type 3I8. These 4 children were probably infected with mutant viruses acquired from hosts previously treated with baloxavir.

Previous studies reported that oseltamivir-resistant viruses were detected in oseltamivir-treated 1–12-year-old children on day >4 after oseltamivir administration (*8*,*9*). During our monitoring for baloxavir-induced mutant influenza viruses, we found that, among baloxavir-treated patients, all but 1 of the mutant viruses were detected 3–6 days after baloxavir administration. One mutant virus was detected the day after baloxavir administration in a 2-year-old child from a family cluster, and this virus possessed a mixture of I38T/I substitutions (50% T and 50% I). This child might have been infected with a mixed population containing mutant and wild-type viruses; this incident suggested possible human-to-human transmission of the mutant influenza A(H3N2) virus encoding the PA I38T substitution.

The 8-month-old infant infected with A/Kanagawa/IC18141/2019 in this study had no exposure to baloxavir before specimen collection. The sibling of this infant, infected with A/Kanagawa/IC18144/2019, was treated with baloxavir for a half day before the infant’s symptoms began. A/Kanagawa/IC18141/2019 and A/Kanagawa/IC18144/2019 viruses possessed the same genomic sequences. In Kanagawa, Japan, H3N2 virus activity was highest in February 2019, and an influenza outbreak occurred in the primary school attended by the sibling. Furthermore, during October 2018–February 2019, baloxavir was supplied to medical institutions that together served ≈5.6 million persons in Japan. These observations suggest 2 possibilities: the infant was infected by the sibling who was infected by another host harboring the virus with the PA I38T substitution, or both children were infected by another host harboring the virus with the PA I38T substitution. The median incubation period of influenza A virus is 1.4 days (*10*), and virus shedding can be detected 1 day before the onset of symptoms (*11*). Considering that the infant did not have much contact with the outside family, the infant acquiring the mutant virus from the sibling is the most likely option.

During our monitoring, 4 of 5 pH1N1 viruses and 26 of 32 H3N2 viruses with the PA I38 substitution were isolated from children <12 years of age. Our results confirm that the frequency of viruses with this mutation is higher in patients <12 years of age than those 12–64 years of age, as previously reported (*5*). Therefore, baloxavir susceptibility of influenza viruses, especially among infected children <12 years of age, should be closely monitored for public health planning purposes and for making clinical recommendations for antiviral drug use.
